# Rapid Assessment of Stroke Severity: Development of a Visual Infarct and Comprehensive Scoring System in Ischemic Rats With Middle Cerebral Artery Occlusion

**DOI:** 10.1111/ejn.70611

**Published:** 2026-07-22

**Authors:** Changqing Mu, Yuchuan Ding, Omar Elmadhoun, Fengwu Li, Xiaokun Geng

**Affiliations:** ^1^ Luhe Institute of Neuroscience Capital Medical University Beijing China; ^2^ Department of Neurology, Beijing Luhe Hospital Capital Medical University Beijing China; ^3^ Department of Neurosurgery Wayne State University School of Medicine Detroit Michigan USA; ^4^ Division of Critical Care Medicine Mayo Clinic Rochester Minnesota USA

**Keywords:** Infarct Score, intraluminal filament model, neurological deficit score, neuroprotective treatment, weight change

## Abstract

Reliable assessment of infarct severity is essential in experimental stroke research, yet commonly used approaches such as 2,3,5‐triphenyltetrazolium chloride (TTC) staining and magnetic resonance imaging require additional tissue processing, specialized equipment, or substantial time and cost. In this study, we developed and validated a rapid visual Infarct Score to determine infarct volume and evaluate neuroprotective efficacy in rat middle cerebral artery occlusion (MCAO) models. A retrospective analysis was performed using data from 315 male Sprague–Dawley rats, including TTC‐derived infarct volume, visual Infarct Score, neurological deficit scores, and body weight changes. The Infarct Score demonstrated the strongest correlation with infarct volume among individual indicators in both nontreatment (Rho = 0.71) and treatment cohorts (Rho = 0.68). We further developed a Comprehensive Score by integrating the Infarct Score with the Longa 5‐point score at 48 h, which showed the highest overall correlation with infarct volume (Rho = 0.75 in both cohorts) and strong discrimination of infarct severity (AUC = 0.90). Interrater reliability analysis demonstrated excellent agreement among independent raters (intraclass correlation coefficient = 0.89; Kendall's *W* = 0.83, both *p* < 0.001). Neuroprotective interventions, including therapeutic hypothermia, nitroglycerin, and remote ischemic conditioning combined with physical exercise, significantly reduced infarct volume, Infarct Score, Comprehensive Score, neurological deficits, and weight loss. These findings indicate that the Infarct Score offers a rapid, practical, and cost‐effective method for infarct assessment, whereas the Comprehensive Score further improves the predictive accuracy and may serve as a useful tool for evaluating stroke severity and treatment outcomes in preclinical studies.

## Graphical abstract

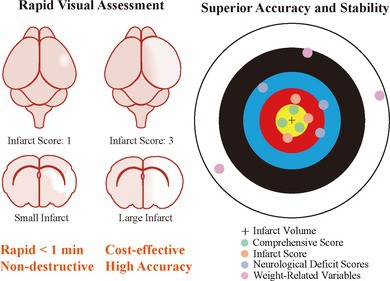

**Abbreviations**
AUC: area under the curve
GTN: nitroglycerin
ICC: intraclass correlation coefficient
MCA: middle cerebral artery
MCAO: middle cerebral artery occlusion
MCAO1h/R: MCAO with 1‐h occlusion followed by reperfusion
MCAO24h/R: MCAO with 24‐h occlusion followed by reperfusion
MCAO2h/R: MCAO with 2‐h occlusion followed by reperfusion
MCAO6h/R: MCAO with 6‐h occlusion followed by reperfusion
MRI: magnetic resonance imaging
pMCAO: permanent middle cerebral artery occlusion
RICE: remote ischemic conditioning combined with exercise
ROC: receiver operating characteristic
SD: Sprague–Dawley
TH: therapeutic hypothermia
tMCAO: transient middle cerebral artery occlusion
TTC: 2,3,5‐triphenyltetrazolium chloride

## 1 Introduction

Ischemic stroke remains a major contributor to global mortality and long‐term neurological disability (Hilkens et al. 2024; Cheng et al. 2025; Kumarapuram et al. 2025). Its pathological nature involves ischemic necrosis of brain tissue due to disrupted blood flow. Animal models, especially the rat middle cerebral artery occlusion (MCAO) model, are critical tools for studying the mechanisms of stroke and evaluating therapeutic interventions (Sokolowski et al. 2023; Qin et al. 2024). Accurate measurement of infarct volume is essential for quantifying the extent of brain tissue damage and validating the efficacy of neuroprotective strategies.

The 2,3,5‐triphenyltetrazolium chloride (TTC) staining method is widely used to quantify infarct volume (Liu et al. 2009; Sanchez‐Bezanilla et al. 2019; Mi et al. 2024). Previous studies have demonstrated that TTC‐stained tissue can remain suitable for subsequent histological, molecular, and biochemical analyses, including hematoxylin–eosin staining, immunohistochemistry, immunofluorescence, Western blotting, and gene expression analyses (Kramer et al. 2010; Li, Yu, and Liang 2018; Li, Bishop, et al. 2018; Omileke et al. 2021). However, TTC‐based infarct assessment requires tissue sectioning and staining procedures, which increase processing time before infarct evaluation. Therefore, a rapid method that allows immediate assessment of stroke severity may still be valuable in certain experimental settings. Magnetic resonance imaging (MRI) offers noninvasive infarct assessment, but high cost, limited availability, and technical requirements restrict its routine application in basic research settings (Boyko et al. 2011; Dou et al. 2025). Neurological deficit scores and weight changes are commonly used to assess stroke severity, but both measures have important limitations. Despite efforts to optimize poststroke behavioral scoring systems, existing methods remain subjective, poorly reproducible, and dependent on training experience (Wen et al. 2017; Yeh et al. 2018). Thus, although simple and inexpensive, neurological scores do not reliably reflect infarct volume (Lee et al. 2016; Ruan and Yao 2020). Moreover, weight loss can be affected by multiple nonneurological factors (Cai et al. 2015). Therefore, these indicators are better considered supplementary measures rather than accurate substitutes for infarct volume (Cai et al. 2015).

To address these limitations, we developed a novel visual assessment method, the Infarct Score. This scoring system is derived from rapid visual inspection of the dorsal brain surface in MCAO rats and evaluates readily observable features, including tissue color, hemispheric swelling, and asymmetry. It offers the advantages of speed, simplicity, minimal cost, and immediate applicability before tissue sectioning or staining. Importantly, this method is intended as a supplementary, nonstaining assessment tool rather than a replacement for TTC‐based infarct quantification, allowing investigators to obtain a rapid estimate of infarct severity while preserving flexibility for subsequent experimental processing.

To develop this system, we analyzed multiple MCAO rat models, incorporating the Infarct Score, neurological deficit scores, weight loss, and TTC‐determined infarct volume. To further enhance predictive accuracy for infarct burden and stroke severity, we constructed a composite Comprehensive Score that integrates the most informative variables. Together, these scoring tools provide a practical, nonstaining, and efficient approach for precise infarct volume estimation in preclinical ischemic stroke research.

## 2 Methods

### 2.1 Animals

Data were retrospectively collected from 315 male Sprague–Dawley (SD) rats (8 to 10 weeks of age) that underwent successful MCAO surgeries at the Luhe Institute of Neuroscience, Capital Medical University, between January 2021 and March 2025. Each rat belonged to an independent study and, according to the respective protocols, received either neuroprotective intervention (*n* = 163) or no treatment (*n* = 152) at all. MCAO was induced using either transient occlusion for 1, 2, 6, or 24 h followed by reperfusion (tMCAO1h/R, tMCAO2h/R, tMCAO6h/R, tMCAO24h/R) or permanent occlusion without reperfusion for 48 h (pMCAO). Among the 315 rats, the model distribution was as follows: tMCAO2h/R (*n* = 269), tMCAO1h/R (*n* = 17), tMCAO6h/R (*n* = 5), tMCAO24h/R (*n* = 3), and pMCAO (*n* = 21). All rats were monitored for 48 h postocclusion before scheduled euthanasia.

For each animal, data collected included body weight, percentage weight change (weight loss), neurological deficits, TTC‐measured infarct volume, and the newly developed Infarct Score. All procedures were approved by the Institutional Animal Care and Use Committee of Capital Medical University and conducted in accordance with institutional and national guidelines. All studies adhered to ARRIVE recommendations for reporting in vivo experiments.

### 2.2 MCAO

This ischemia model was previously described by our group (Wang et al. 2012; Li et al. 2019; Li et al. 2021). Rats were anesthetized in a chamber with a mixture of 1%–3% isoflurane and 70% nitrous oxide with 30% oxygen for a short period. Anesthesia was then maintained using a facemask with 1% isoflurane delivered from a calibrated precision vaporizer. To minimize variability between rats, poly‐L‐lysine‐coated intraluminal nylon (4.0) sutures were used and advanced from the external carotid artery into the internal carotid artery until their tip occluded the MCA at its origin. For tMCAO, the filament was retracted after 1, 2, 6, or 24 h of ischemia to allow reperfusion, and the rats were euthanized 48 h postocclusion. For pMCAO, the monofilament was left in place for 48 h until euthanasia. Rectal temperature was continuously monitored with a rectal probe and maintained at 37.5°C ± 0.5°C using a temperature controller connected to a heat lamp until the animal regained consciousness. Successful MCAO induction was verified by postoperative neurological deficit assessment, endpoint TTC‐confirmed infarction, and, when available, cerebral perfusion monitoring. All included animals demonstrated postoperative neurological deficits, defined as a Longa 5‐point score ≥ 1, and infarct formation on endpoint TTC staining. In experiments using laser Doppler flowmetry or laser speckle imaging, successful occlusion was defined as a reduction in cerebral blood flow of > 70% from baseline (Ran et al. 2018; Zhang et al. 2026). Animals were excluded from analysis if they showed no postoperative neurological deficit, no infarction on TTC staining, insufficient cerebral blood flow reduction or inadequate reperfusion when perfusion monitoring was performed, death before the scheduled endpoint, or technical complications such as hemorrhage.

### 2.3 Neuroprotective Interventions

The neuroprotective treatments administered either before or after stroke induction included hypothermia (TH), nitroglycerin (GTN), and remote ischemic conditioning combined with exercise (RICE). Detailed protocols for these interventions have been described previously by us (Wang et al. 2021; Guo et al. 2022; Jiang, Ding, Li, et al. 2024; Jiang, Ding, Wang, et al. 2024), each demonstrating neuroprotective efficacy in ischemic stroke models. Additional neuroprotective treatments from individual studies also contributed data to this analysis, including intraperitoneal hypothermia, splenectomy, and combined splenectomy with omentectomy. These additional treatments are part of ongoing research efforts, and their detailed findings have not yet been published.

### 2.4 Data Collection for Weight and Weight Change

Body weight was measured before MCAO surgery (baseline) and again at 24 and 48 h after surgery. Weight change (weight loss) was expressed as a percentage of baseline weight, calculated using the following formula:

Weight change (%) = [Body weight at time point after stroke/Baseline weight] × 100%.

### 2.5 Data Collection for Neurological Deficit Score

The neurological deficits were assessed using the modified scoring systems (5 and 12 points) developed by Longa et al. (1989) and Belayev et al. (1996), respectively. Assessments were performed before surgery (baseline), during 2‐h MCA occlusion (for tMCAO and pMCAO), and at 24‐ and 48‐h postsurgery.

### 2.6 Measurement of Infarct Volumes With TTC Staining

At 48 h after MCAO, rats were euthanized, and the brains were rapidly removed. Brains were sectioned into 2‐mm coronal slices using a brain matrix and stained with TTC (Sigma‐Aldrich, St. Louis, MO, USA). Infarct volume was calculated using an indirect method to minimize errors caused by edema (Lin et al. 1993; Wang et al. 2012). The infarct cross‐sectional area of each brain slice was measured using ImageJ software (ImageJ version 1.53t, National Institutes of Health, Bethesda, MD, USA). The infarct volume (%) for each brain slice was calculated as [(total volume of the left hemisphere − noninfarcted volume of the right hemisphere)/(total volume of the left hemisphere)] × 100%. Finally, the total infarct volume for each brain was determined, and the average infarct area across all slices was calculated.

### 2.7 Determination of Infarct Score

Infarct severity at 48 h after MCAO was evaluated using a visual dorsal‐brain assessment, termed the Infarct Score, by an observer blinded to all group assignments. Scoring was based on side‐by‐side comparison of the ipsilateral and contralateral hemispheres. Two macroscopic features were assessed: (1) the extent of pale or white ischemic tissue in the ipsilateral hemisphere and (2) the degree of hemispheric swelling.

**FIGURE 1:**
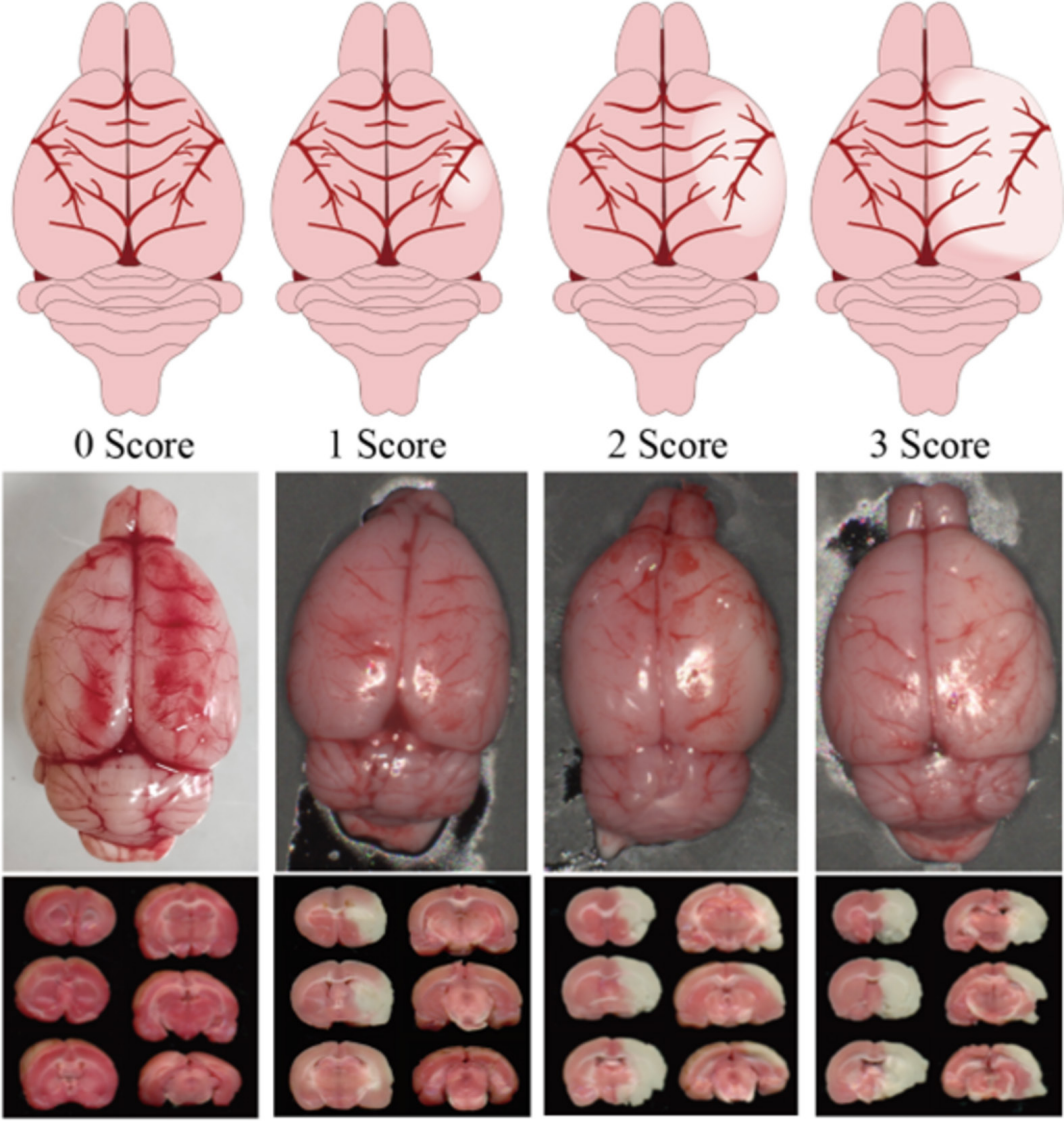
The criteria diagram of Infarct Score. Dorsal brain inspection at 48 h post‐MCAO was used to assign an Infarct Score from 0 to 3 based on the extent of pale ischemic tissue and ipsilateral hemispheric swelling. A score of 0 indicates no visible lesion, 1 indicates a small MCA territory lesion, 2 indicates whitening of the entire MCA territory with mild swelling, and 3 indicates extensive involvement of the MCA and watershed regions with marked swelling. Representative drawings and corresponding brain images illustrate each score category.

Brains were assigned a score from 0 to 3 according to the predefined criteria summarized in Table 1, which describe infarct extent, hemispheric involvement, and swelling. The scoring system captures increasing severity from no visible lesion (0) to extensive whitening and marked swelling (3). Representative illustrations and corresponding TTC images are shown in Figure 1.

**TABLE 1:** Criteria for Infarct Score assessment.

Score	Extent of white (ischemic) region	Swelling of ipsilateral hemisphere	Approximate hemisphere involvement
0	No visible white region compared with contralateral side	None	0%
1	Small white region limited to MCA territory	None	~33%
2	Entire MCA territory appears white	Mild swelling	~50%
3	Whitening extends into MCA + watershed areas	Marked swelling	~67%

*Note:* Scoring criteria for the visual assessment of cerebral infarct severity following ischemic stroke, based on the extent of ischemic whitening, degree of ipsilateral hemispheric swelling, and estimated percentage of hemisphere involvement.

### 2.8 Construction and Validation of the Comprehensive Score

For accurate prediction of infarct volume in ischemic brain tissue, a Comprehensive Score was constructed by combining the Infarct Score with the next best performing predictor of infarct volume. The latter variable was rescaled to a 0–3 point system consistent with the Infarct Score before integration into the final composite measure. In the nontreatment cohort, the correlation between the Comprehensive Score and infarct volume was calculated.

To evaluate the stability and generalizability of the Comprehensive Score, we performed correlation analysis between the Comprehensive Score and all 13 collected variables and infarct volume in the neuroprotection treatment cohort. The treatment cohorts were included not to re‐evaluate the efficacy of these interventions but to test whether the proposed scoring system could reliably reflect TTC‐derived infarct volume across a wider range of infarct severities and treatment responses. By comparing the strength of correlation between the Comprehensive Score and infarct volume with those of all other variables, we assessed whether the predictive performance of the Comprehensive Score remained superior in another dataset. Additionally, by comparing the correlation of the Comprehensive Score between the nontreatment and treatment cohorts, we evaluated the stability of the score across different groups.

### 2.9 Linear Regression Analysis and Cross‐Validation for Each Variable

To evaluate the predictive performance of the Infarct Score and Comprehensive Score for infarct volume, linear regression analysis was performed on the integrated dataset using 10‐fold cross‐validation. The analysis was based on the integrated data of the no‐treatment and treatment groups. The average *R* ^2^ value was used to assess the overall model fit, whereas the standard deviation of *R* ^2^ across folds was used to measure the stability of the model during cross‐validation.

### 2.10 Application of Infarct Score and Comprehensive Score in Assessing Infarct Severity

To further determine whether the Infarction Score and the Comprehensive Score can reliably classify infarct severity, we evaluated their discriminative performance using receiver operating characteristic (ROC) analysis. All MCAO rats, including both untreated and treated animals, were pooled and graded into small‐infarct and large‐infarct groups according to the median TTC‐measured infarct volume. ROC curves were generated for each variable using the pROC package, and the area under the curve (AUC) was calculated to quantify classification accuracy. This analysis enabled direct comparison of the diagnostic utility of individual variables and the composite scoring system. For the variable with the highest AUC, the Youden index was used to identify the optimal discriminatory threshold, thus establishing an objective cutoff value for differentiating infarct severity.

### 2.11 Evaluation of Treatment Effects by Differential Analysis

To determine whether the Infarct Score and Comprehensive Score accurately capture treatment‐induced reductions in infarct burden, we compared each variable between the treatment group and the nontreatment group. In addition to statistical testing, we calculated fold change, defined as the ratio of the mean value of a variable in the treatment group to that in the nontreatment group. By comparing the fold changes of individual variables with the fold change of TTC‐measured infarct volume, we assessed the ability of each indicator to reflect the extent of neuroprotective benefit. Variables whose fold change patterns closely paralleled that of infarct volume were considered more accurate and sensitive markers of treatment efficacy.

### 2.12 Statistical Analysis

All statistical analyses and visualizations were performed using R software (version 4.2.1). All variables are presented as means ± standard error (Mean ± SE), Shapiro–Wilk normality tests and Levene's tests for homogeneity of variance were performed on all continuous variables. For comparisons between two groups, an independent *t*‐test was used if the data met normality and homogeneity of variance assumptions; otherwise, the nonparametric Mann–Whitney *U* test was applied. Spearman's rank correlation was used for correlation analysis. The correlation strength was classified as follows based on the absolute value: 0.01–0.19 (no or negligible relationship), 0.20–0.29 (weak relationship), 0.30–0.39 (moderate relationship), 0.40–0.69 (strong relationship), and 0.70 or greater (very strong relationship) (Hu et al. 2016). The significance level was set at a two‐sided *p*‐value of 0.05. For correlation analyses, *p*‐values were adjusted using the Bonferroni method to control the false discovery rate. Previous studies have shown a significant correlation between the Longa score and infarct volume (Rho = 0.53, *p* < 0.05) (Hu et al. 2015). Interrater reliability of the Infarct Score was assessed using three independent raters. Agreement among raters was evaluated using Kendall's coefficient of concordance (Kendall's *W*) and intraclass correlation coefficients (ICC). To explore the correlation between the variables collected in this study and infarct volume, we conducted a power calculation based on these previous results. The results indicated that to meet the criteria of a 5% significance level and 80% power, at least 28 samples were required. The power calculation was performed using PASS software (version 2021). Visualizations were generated using the ggplot2 package.

## 3 Results

### 3.1 Correlation Between Variables and Infarct Volume

**FIGURE 2:**
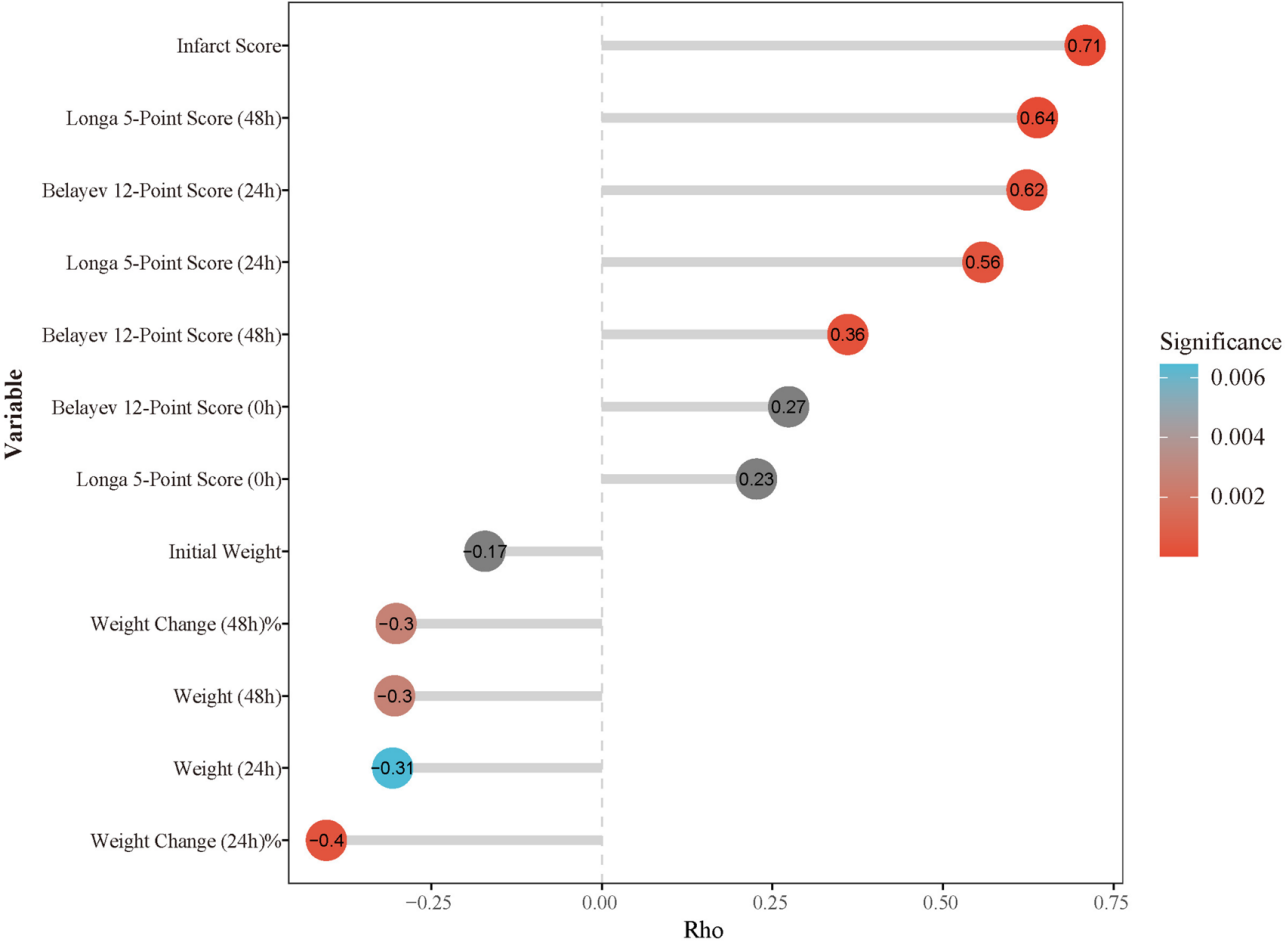
Correlation analysis of 12 variables with infarct volume. Correlations between individual variables and TTC measured infarct volume were assessed using Spearman rank correlation coefficients, reported as Rho. The color gradient represents statistical significance. The Infarct Score at 48 h poststroke showed the strongest correlation with infarct volume (Rho = 0.71), followed by the Longa 5‐point score at 48 h (Rho = 0.64). Neurological deficit scores at 24 and 48 h demonstrated weaker associations. Among weight‐related variables, weight change at 24 h showed the strongest correlation with infarct volume (Rho = −0.40).

Correlation analyses revealed that 12 variables were significantly associated with TTC‐measured infarct volume (Figure 2 and Table 2). Among all markers examined, the Infarct Score at 48 h poststroke showed the strongest and most robust association (Rho = 0.71, adjusted *p*‐value = 9.58 × 10^−14^), representing the only very strong correlation in the dataset. This finding identifies the Infarct Score as the most accurate single macroscopic indicator of infarct burden in the MCAO model.

**TABLE 2:** Correlation analysis of 12 variables with infarct volume.

Variable	Rho	*p*	Adjusted *p*
Infarct Score	0.71	7.98 × 10–15	9.58 × 10–14
Longa 5‐point score (48 h)	0.64	1.44 × 10–18	1.73 × 10–17
Belayev 12‐point score (24 h)	0.62	1.07 × 10–4	1.29 × 10–3
Longa 5‐point score (24 h)	0.56	1.79 × 10–4	2.15 × 10–3
Belayev 12‐point score (48 h)	0.36	6.51 × 10–5	7.81 × 10–4
Belayev 12‐point score (0 h)	0.27	0.10	1.00
Longa 5‐point score (0 h)	0.23	0.18	1.00
Initial weight	−0.17	0.08	0.93
Weight change (48 h) %	−0.30	1.78 × 10–3	0.02
Weight (48 h)	−0.30	1.63 × 10–3	0.02
Weight (24 h)	−0.31	4.84 × 10–3	0.06
Weight change (24 h) %	−0.40	1.54 × 10–4	1.85 × 10–3

*Note:* This table presents the Spearman correlation coefficients (Rho), *p*‐values, and adjusted *p*‐values for 12 variables associated with TTC‐measured infarct volume. Variables are ordered by strength of correlation. The Infarct Score showed the strongest association (Rho = 0.71, adjusted *p* = 9.58 × 10^−14^), followed by the Longa 5‐point score at 48 h. Other variables demonstrated progressively weaker associations, indicating variable predictive utility.

Neurological deficit scores also demonstrated meaningful but comparatively weaker relationships with infarct volume. The Longa 5‐point score (48 h) (Rho = 0.64), Longa 5‐point score (24 h) (Rho = 0.56), and Belayev 12‐point score (24 h) (Rho = 0.62) each exhibited strong positive correlations, supporting their value as functional readouts of poststroke injury severity. However, none matched the predictive strength of the Infarct Score, suggesting that behavioral performance reflects neurological dysfunction but does not provide a precise estimate of tissue loss.

As expected, neurological assessments obtained immediately after stroke induction showed no correlation with infarct volume, consistent with early‐phase variability and the lack of established tissue injury at that time point.

Weight‐related variables showed inconsistent and generally weaker associations. Initial body weight and weight at 24 h were not significantly correlated with TTC‐measured infarct volume (adjusted *p* = 0.93 and 0.06, respectively). In contrast, weight at 48 h (Rho = −0.30) and weight loss at 48 h (Rho = −0.30) demonstrated moderate negative correlations, indicating that larger infarctions were associated with greater metabolic decline. Notably, weight loss at 24 h showed the strongest weight‐related correlation (Rho = −0.40), suggesting early metabolic changes may partially reflect evolving infarct severity.

Taken together, these results demonstrate that the Infarct Score provides the most reliable single surrogate for infarct volume, outperforming neurological assessments and systemic indicators. Its strong correlation, simplicity, and nonstaining nature highlight its value as a rapid assessment tool in preclinical ischemic stroke research.

### 3.2 Construction and Verification of Comprehensive Scoring System

**FIGURE 3:**
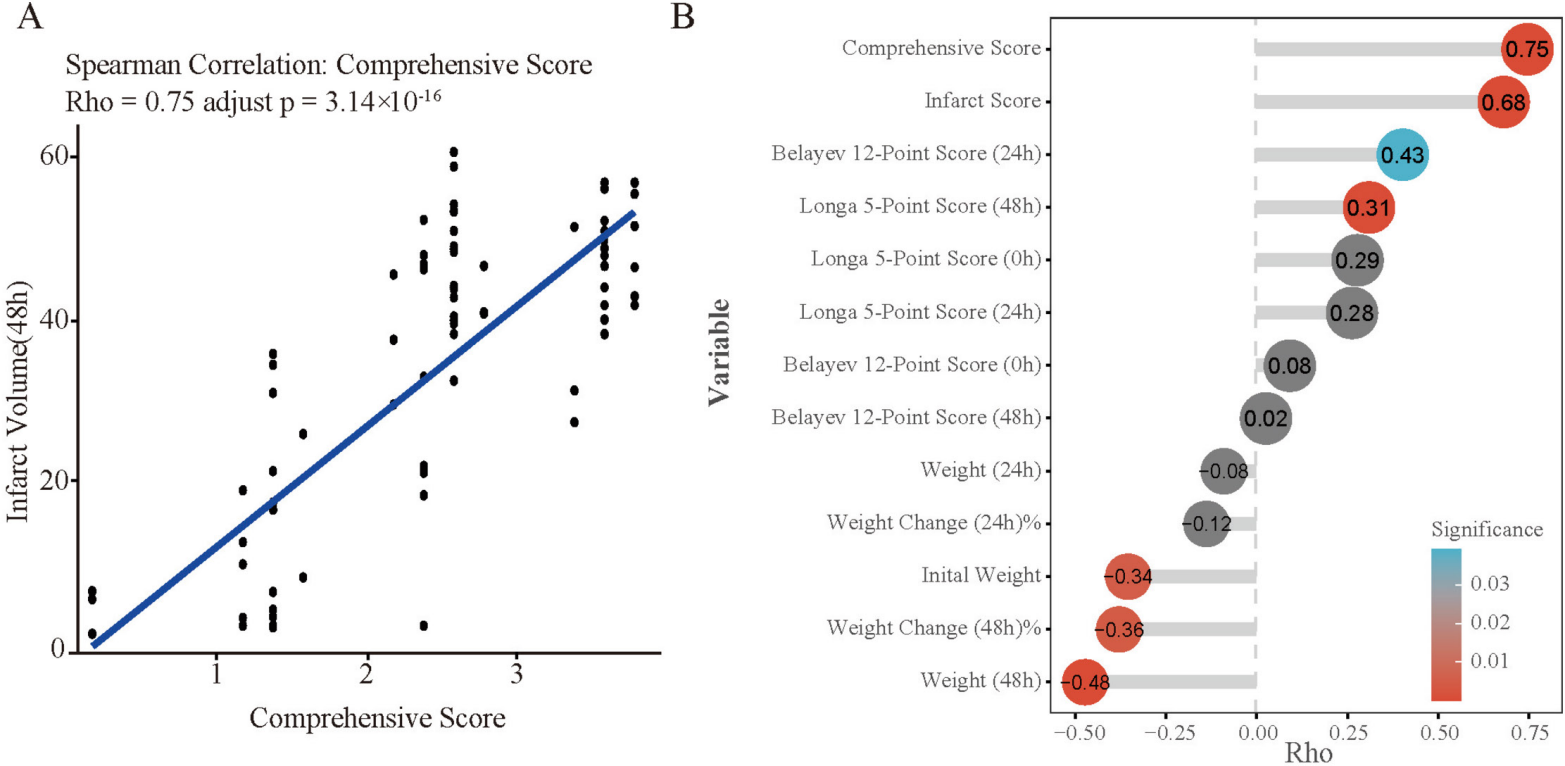
Correlation analysis of the Comprehensive Score and other variables in nontreatment and treatment groups. (A) In untreated animals, the Comprehensive Score demonstrated the strongest association with infarct volume (Rho = 0.75), exceeding all other evaluated variables. (B) In treated animals, the Comprehensive Score remained the strongest predictor of infarct volume (Rho = 0.75). The Infarct Score ranked second (Rho = 0.68). The Longa 5‐point score at 48 h retained a moderate association (Rho = 0.31), whereas other neurological scores showed no significant correlation. Weight at 48 h (Rho = −0.48) and weight change at 48 h (Rho = −0.36) showed moderate negative associations.

To enhance infarct volume prediction beyond any single indicator, we integrated the Infarct Score with the next most strongly correlated variable, Longa 5‐point score (48 h), to generate a Comprehensive Score. In the nontreatment cohort, the Comprehensive Score exhibited the strongest positive relationship with infarct volume (Rho = 0.75, adjusted *p* = 1.57 × 10^−16^), surpassing all other 12 variables evaluated (Figure 3A).

Validation analysis in the treatment group further confirmed its superior predictive performance. The Comprehensive Score again showed the highest association with infarct volume (Rho = 0.75, adjusted *p* = 9.99 × 10^−7^), whereas the Infarct Score (Rho = 0.68, adjusted *p* = 6.09 × 10^−7^) ranked second, showing only a slight reduction in strength relative to untreated animals (Figure 3B and Table 3).

**TABLE 3:** Correlation analysis of Comprehensive Score and 12 variables with infarct volume.

Variable	Rho	*p*	Adjusted *p*
Comprehensive Score	0.75	7.68 × 10^−8^	9.99 × 10^−7^
Infarct Score	0.68	4.69 × 10^−8^	6.09 × 10^−7^
Belayev 12‐point score (24 h)	0.43	0.02	0.28
Longa 5‐point score (48 h)	0.31	1.06 × 10^−4^	1.38 × 10^−3^
Longa 5‐point score (0 h)	0.29	0.09	1.00
Longa 5‐point score (24 h)	0.28	0.10	1.00
Belayev 12‐point score (0 h)	0.08	0.64	1.00
Belayev 12‐point score (48 h)	0.02	0.80	1.00
Weight (24 h)	−0.08	0.65	1.00
Weight change (24 h) %	−0.12	0.49	1.00
Weight change (48 h) %	−0.36	1.70 × 10^−3^	0.02
Weight (48 h)	−0.48	1.45 × 10^−5^	1.88 × 10^−4^

*Note:* This table presents Spearman correlation coefficients (Rho), *p*‐values, and adjusted *p*‐values for 12 variables evaluated in the treatment group. The Comprehensive Score demonstrated the strongest association with infarct volume (Rho = 0.75, adjusted *p* = 9.99 × 10^−7^), followed by the Infarct Score (Rho = 0.68, adjusted *p* = 6.09 × 10^−7^), confirming the superior predictive performance of the composite scoring system. Neurological deficit scores showed weaker correlations under treatment conditions.

Regarding the neurological assessments, Longa 5‐point score (48 h) (Rho = 0.31, adjusted *p* = 1.38 × 10^−3^) retained a significant but weaker association under treatment conditions. Other neurological deficit scores at all time points lost their correlations with infarct volume once neuroprotective treatments were introduced, suggesting that treatment effects differentially influence functional and structural outcomes.

Weight‐related variables also behaved differently between groups. In the treatment group, weight (48 h) (Rho = −0.48, adjusted *p*‐value = 1.88 × 10^−4^) and weight Change (48 h) % (Rho = −0.36, adjusted *p* = 0.02) exhibited moderate negative associations with infarct volume, whereas weight (24 h) and weight change (24 h) % showed no significant relationships (adjusted *p* = 1.00 for both).

To address potential confounding from model heterogeneity, a sensitivity analysis restricted to the predominant tMCAO2h/R subgroup was performed. The results remained consistent with the primary analysis. In particular, both the Infarct Score (Rho = 0.71, adjusted *p* < 0.001) and the Comprehensive Score (Rho = 0.75, adjusted *p* < 0.001) maintained strong correlations with TTC‐derived infarct volume (Table S1).

Collectively, these results validate the Comprehensive Score as the most stable and powerful predictor of infarct volume across both untreated and treated conditions, outperforming individual neurological or weight‐based indicators and demonstrating strong generalizability across experimental settings.

### 3.3 Cross‐Validation Prediction Performance Evaluation

To assess the predictive strength of each variable for estimating infarct volume, we performed linear regression modeling with 10‐fold cross validation across the combined dataset of treated and untreated animals. This analysis provided two key metrics for each variable: (1) predictive accuracy, quantified by the average *R* ^2^ value across folds, and (2) model stability, determined by the variability (standard deviation) of *R* ^2^ values across folds.

**FIGURE 4:**
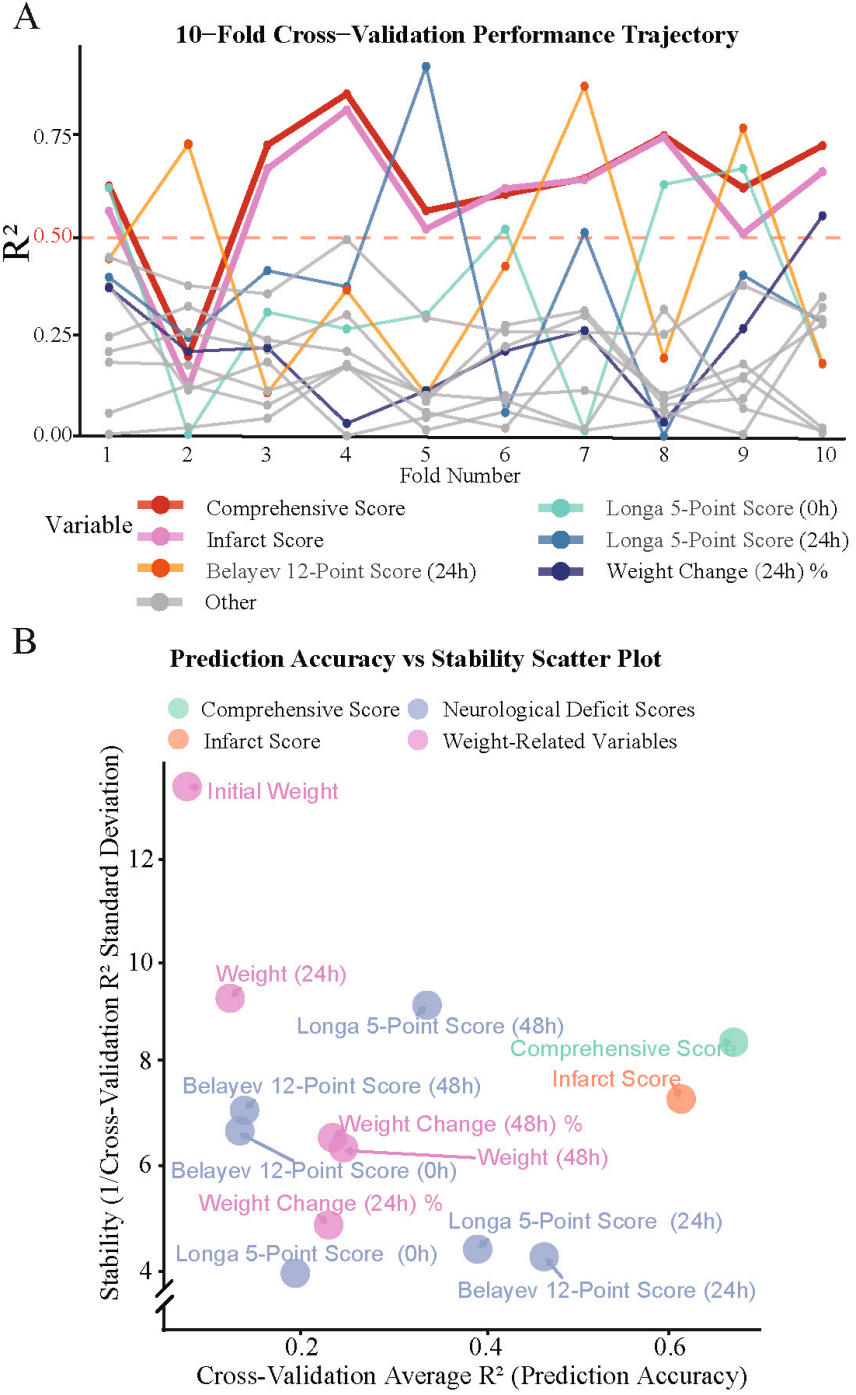
Performance of variables for infarct volume prediction using 10‐fold cross‐validation. (A) 10‐Fold cross‐validation performance trajectory. The plot shows the *R* ^2^ values for each variable across the 10‐fold cross‐validation, with the dashed horizontal line indicating the *R* ^2^ threshold of 0.5. Variables that consistently exceeded this threshold in 9 of 10 folds (Comprehensive Score and Infarct Score) demonstrated strong and stable predictive capacity for infarct volume. Other variables, such as the Belayev 12‐point score (24 h) and Longa 5‐point score (0 h), achieved *R* ^2^ > 0.5 in only a few folds (1–5 of 10 folds), indicating weaker predictive performance and reliability. Variables not exceeding the threshold in any fold are categorized as having poor explanatory power and are represented in gray. (B) Prediction accuracy versus stability scatter plot. This scatter plot illustrates the relationship between prediction accuracy (average *R* ^2^ across folds, on the *x*‐axis) and stability (inverse of the standard deviation of *R* ^2^ values, on the *y*‐axis). The variable positioned near the upper right quadrant indicates its high accuracy and stability. The initial weight variable is highly stable but has no meaningful correlation with infarct volume. When excluding the initial weight variable, both the Comprehensive Score and Infarct Score would be positioned more clearly in the upper right corner. This analysis underscores that the Comprehensive Score and Infarct Score are the most suitable indicators for infarct volume prediction, demonstrating both robust accuracy and stability.

As shown in Figure 4A, only the Comprehensive Score and Infarct Score consistently achieved *R* ^2^ values greater than 0.5 in 9 out of 10 folds, indicating their strong and reliable explanatory capacity for infarct volume. In contrast, other variables, including neurological scores and weight‐related measures, rarely exceeded the *R* ^2^ > 0.5 threshold. The Belayev 12‐point score (24 h) exceeded this threshold in only three folds, and the Longa 5‐point score at 0 and 24 h exceeded it in just two folds each. All remaining variables failed to reach the threshold in any fold, indicating limited predictive utility.

A scatterplot in Figure 4B further illustrated the relationship between prediction accuracy and stability. The Comprehensive Score achieved the highest overall accuracy while maintaining low variability across folds, positioning it as the most reliable predictor among all variables examined. The Infarct Score exhibited similarly strong performance, though slightly less stable than the Comprehensive Score. In contrast, most neurological and weight‐related variables were grouped in regions of lower accuracy and greater variability, indicating reduced performance in both prediction and consistency.

Although certain variables, such as initial weight, weight (24 h), and Longa 5‐point score (48 h), demonstrated relatively stable *R* ^2^ values, their predictive accuracy remained substantially lower than that of the Comprehensive Score and Infarct Score. This finding underlines that stability alone does not compensate for low predictive strength.

Taken together, the cross‐validation analysis confirms that the Comprehensive Score provides the best combination of predictive accuracy and model stability for estimating TTC‐measured infarct volume across heterogeneous experimental conditions. The Infarct Score also performs well, reinforcing its value as a rapid structural assessment tool. However, no neurological or systemic variable approached the predictive performance of these two scoring systems.

### 3.4 Infarct Severity Classification and Evaluation Using ROC Curves and Youden Index

**FIGURE 5:**
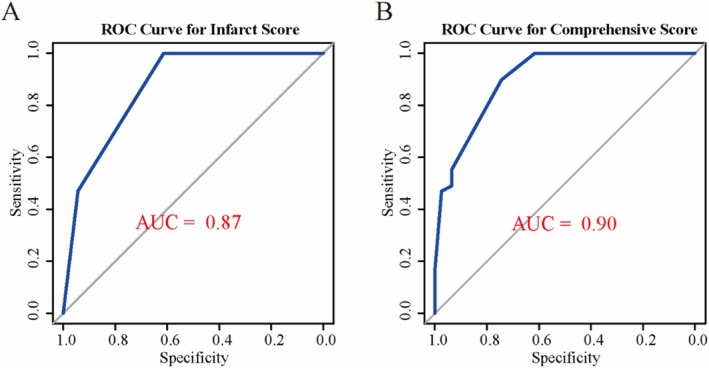
ROC curve analysis for assessing infarct volume severity. (A) The Infarct Score showed good discrimination between large and small infarcts with an area under the curve of 0.87. (B) The Comprehensive Score demonstrated superior performance with an area under the curve of 0.90, confirming its accuracy in infarct severity classification.

To determine the ability of each variable to discriminate between different levels of infarct severity, all MCAO rats, regardless of treatment status, were divided into small‐infarct and large‐infarct groups based on the median TTC‐measured infarct volume (40.21%). ROC curves analysis was then performed for each variable, and the AUC was calculated to quantify classification accuracy (Figure 5).

The Comprehensive Score demonstrated the highest discriminatory performance (AUC = 0.90), indicating excellent accuracy in distinguishing large infarcts from small ones. The Infarct Score (AUC = 0.87) ranked second, also achieving high classification ability and confirming its strong structural predictive values. Other variables, including neurological scores and weight‐related measures, showed notably lower AUC values (Table 4), consistent with their weaker and more variable associations with infarct volume observed in prior analyses.

**TABLE 4:** Areas under the curve (AUC) for all variables in the ROC analysis discriminating large versus small infarcts.

Variable	AUC
Comprehensive Score	0.90
Infarct Score	0.87
Belayev 12‐point score (24 h)	0.82
Longa 5‐point score (48 h)	0.75
Longa 5‐point score (24 h)	0.75
Weight change (48 h) %	0.74
Weight (48 h)	0.71
Weight change (24 h) %	0.70
Belayev 12‐point score (48 h)	0.69
Weight (24 h)	0.61
Belayev 12‐point score (0 h)	0.59
Initial weight	0.56
Longa 5‐point score (0 h)	0.52

*Note:* This table presents area under the curve values for all variables used to discriminate between large and small infarcts in MCAO rats. The Comprehensive Score demonstrated the highest discriminatory performance (area under the curve = 0.90), followed by the Infarct Score (area under the curve = 0.87), indicating excellent classification accuracy. Neurological and weight‐related variables showed moderate performance, whereas acute neurological scores and initial body weight exhibited limited discriminatory capacity.

For the best‐performing variables, the Comprehensive Score, we further computed the Youden index to determine the optimal cutoff for infarct severity classification. The optimal threshold was identified as 2.5, providing a practical, objective reference point for categorizing ischemic injury severity in experimental stroke studies. A Comprehensive Score greater than 2.5 corresponds to severe infarction, whereas values below this threshold indicate mild to moderate infarct burden.

These findings reinforce the superior predictive capacity of the Comprehensive Score and support its utility as a robust, quantitative tool for infarct stratification in preclinical research.

### 3.5 Comparison of Variables Between Treated and Untreated Groups

To evaluate whether neuroprotective interventions produced measurable improvements across structural, neurological, and systemic indicators, we compared all variables between the treatment and nontreatment groups under the MCAO 2 h reperfusion condition.

**FIGURE 6:**
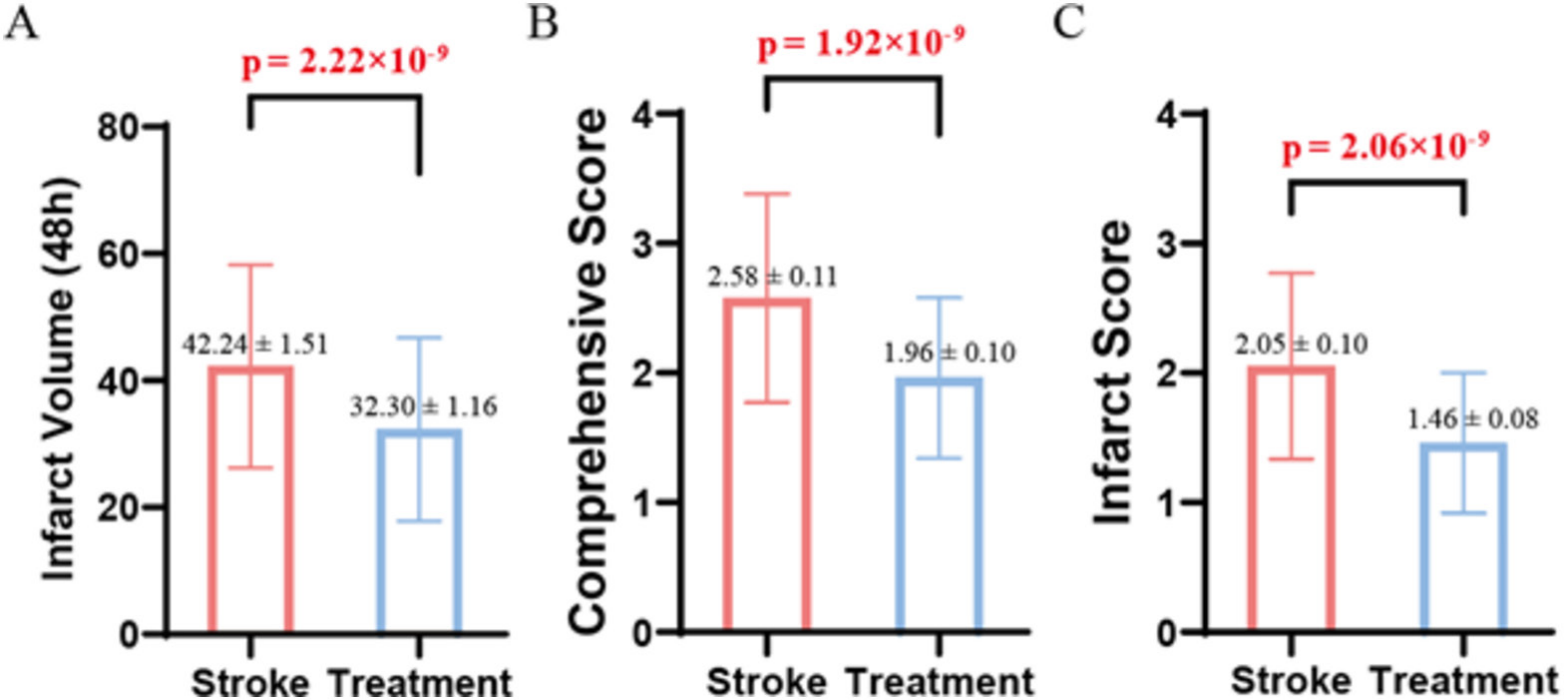
Comparison of infarct volume, Comprehensive Score, and Infarct Score between treatment and no treatment groups at 48 h post–ischemic stroke. (A) Infarct volume at 48 h poststroke. Treatment significantly reduced infarct volume (No Treatment: 42.24 ± 1.51; Treatment: 32.30 ± 1.16; *p* = 2.22 × 10^−9^), confirming neuroprotective efficacy. (B) Comprehensive Score at 48 h poststroke. The Comprehensive Score was significantly lower in the treatment group (No Treatment: 2.58 ± 0.11; Treatment: 1.96 ± 0.10; *p* = 1.92 × 10^−3^), demonstrating sensitivity to treatment‐induced reductions in infarct burden. (C) Infarct Score at 48 h poststroke. The Infarct Score was also significantly reduced in treated animals (No Treatment: 2.05 ± 0.10; Treatment: 1.46 ± 0.08; *p* = 2.06 × 10^−5^), supporting its reliability as a structural marker of infarct severity following neuroprotective treatment.

As expected, TTC‐measured infarct volume was significantly reduced in the treatment group (*p* = 2.22 × 10^−9^), confirming the overall neuroprotective efficacy of the interventions. Both the Comprehensive Score (*p* = 1.92 × 10^−3^) and Infarct Score (*p* = 2.06 × 10^−5^) at 48 h were likewise significantly lower in treated animals (Figure 6), demonstrating that these scoring systems reliably tracked treatment‐induced reductions in infarct burden.

**FIGURE 7:**
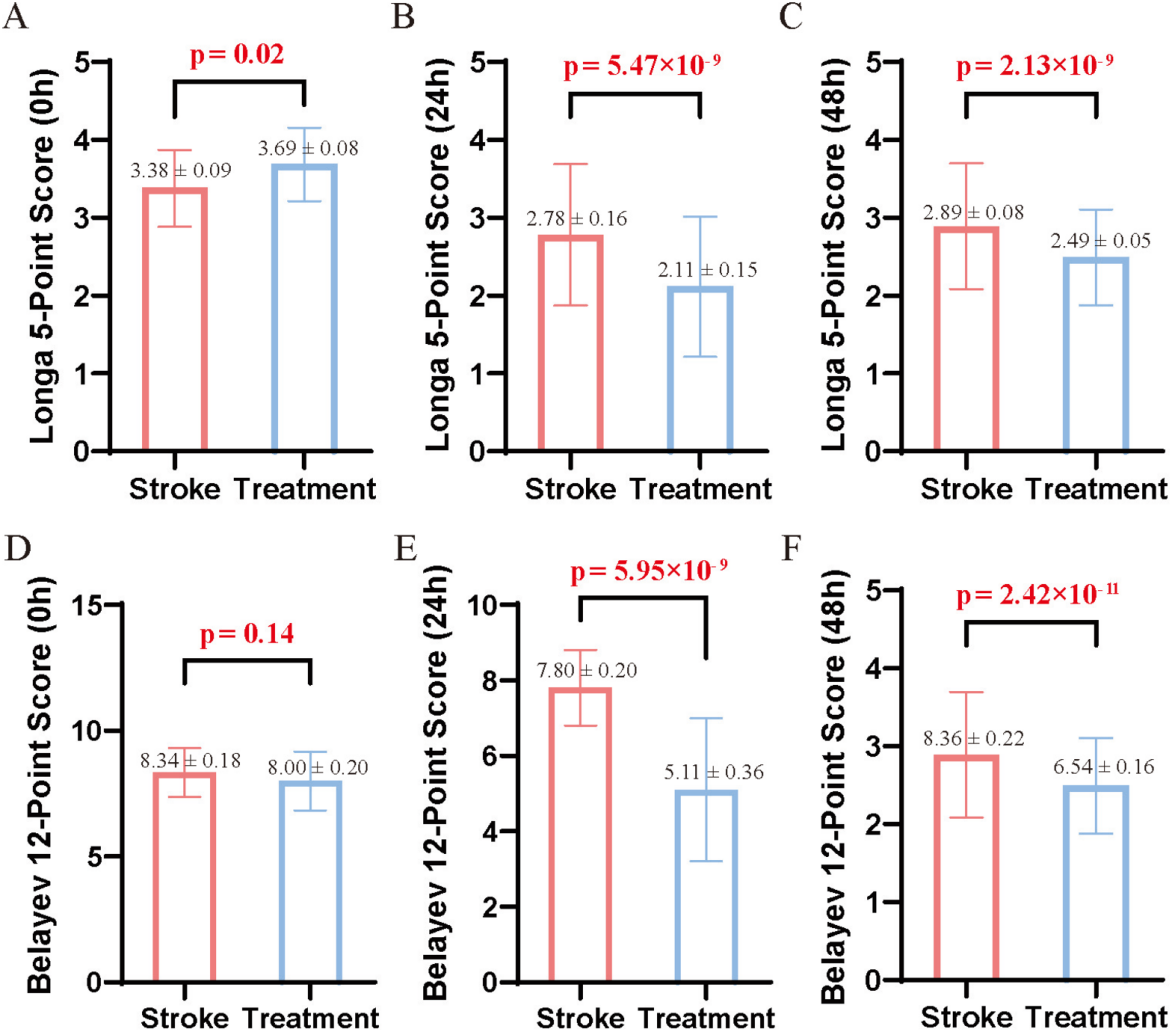
Comparison of neurological deficit scores at multiple time points with and without treatment. (A) Longa 5‐point score at 0 h. At the acute time point, the Longa 5‐point score was slightly but significantly higher in the treatment group (3.69 ± 0.08) compared with the no treatment group (3.38 ± 0.09; *p* = 0.02). This early difference is consistent with the lack of correlation between acute neurological assessments and final infarct volume, indicating that scores obtained immediately after stroke induction are influenced by transient physiological factors rather than tissue injury. (B) Longa 5‐point score at 24 h. The Longa 5‐point score at 24 h poststroke was significantly lower in the treatment group (2.11 ± 0.15) compared with the nontreatment group (2.78 ± 0.16; *p* = 5.47 × 10^−9^), indicating partial functional recovery. (C) Longa 5‐point score at 48 h. The Longa 5‐point score at 48 h also showed a significant reduction in the treatment group (2.49 ± 0.05) compared with the nontreatment group (2.89 ± 0.08; *p* = 2.13 × 10^−9^), reflecting continued functional improvement. (D) Belayev 12‐point score at 0 h. The Belayev 12‐point score at 0 h poststroke did not differ significantly between treatment (8.00 ± 0.20) and nontreatment groups (8.34 ± 0.18; *p* = 0.14), consistent with early phase variability in neurological assessments. (E) Belayev 12‐point score at 24 h. At 24 h poststroke, the treatment group showed significantly lower Belayev 12‐point scores (5.11 ± 0.36) compared with the nontreatment group (7.80 ± 0.20; *p* = 5.95 × 10^−9^), indicating substantial functional improvement. (F) Belayev 12‐point score at 48 h. The Belayev 12‐point score at 48 h was also significantly lower in the treatment group (6.54 ± 0.16) compared with the nontreatment group (8.36 ± 0.22; *p* = 2.42 × 10^11^), reflecting sustained neurological recovery.

Neurological deficit scores exhibited a time‐dependent pattern. At the acute time point (0 h), the Belayev 12‐point score did not differ between treatment and nontreatment groups (*p* = 0.14), whereas the Longa 5‐point score showed a small but statistically significant difference (*p* = 0.02), with slightly higher scores in treated animals (Figure 7A). This early difference is consistent with the previously observed lack of correlation between acute neurological assessments and final infarct volume, indicating that neurological scores obtained immediately after stroke induction are influenced by transient physiological factors and do not reliably reflect treatment efficacy or ultimate tissue injury. By 24 and 48 h poststroke, both neurological scoring systems exhibited significant improvement in the treatment group (Figure 7B–F), indicating partial functional recovery that was consistent with reduced brain infarction.

**FIGURE 8:**
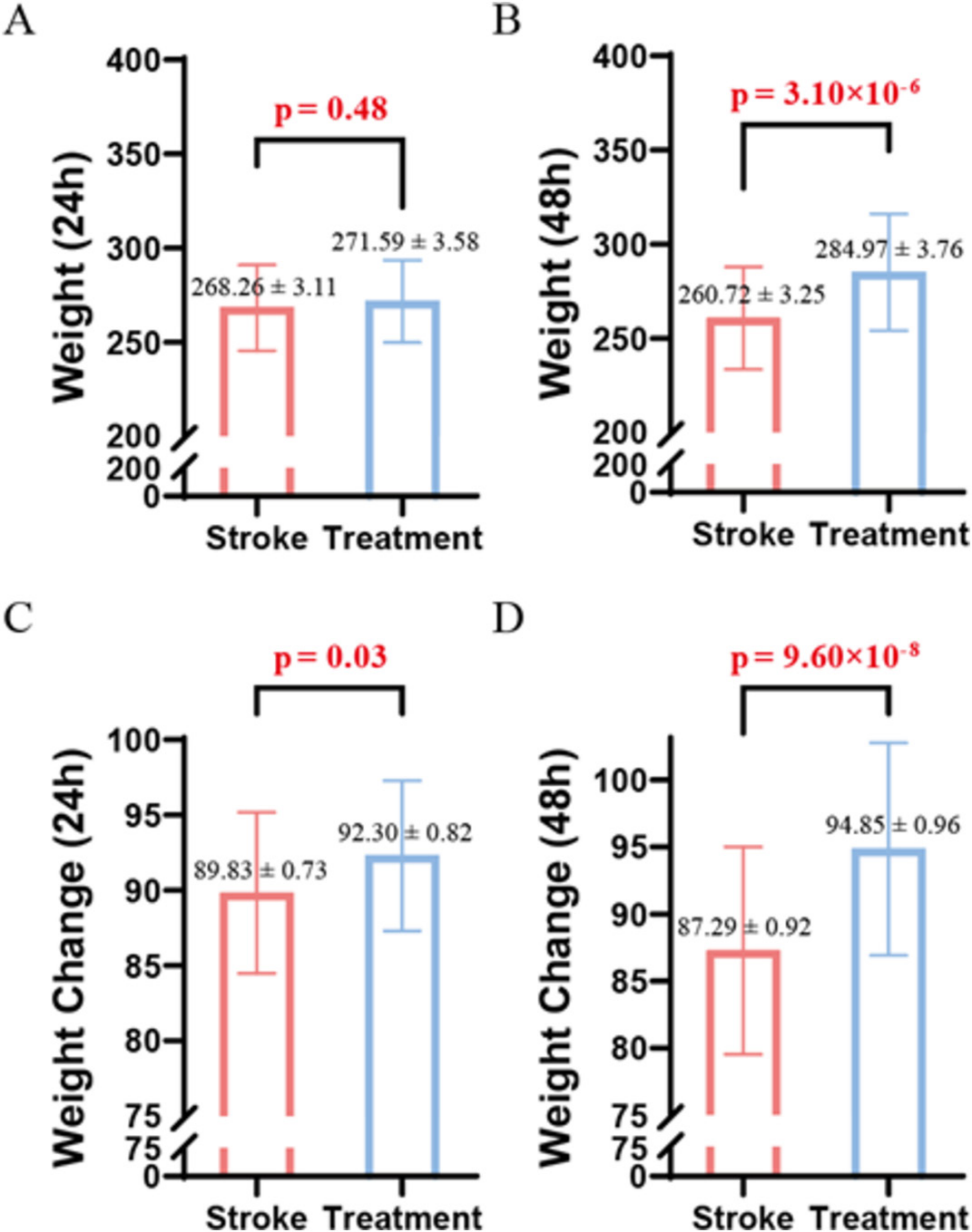
Comparison of weight and weight change percentage at multiple time points with and without treatment. Weight‐related measures showed no group differences early after stroke but significant preservation of body weight and reduced weight loss at 48 h in treated animals. (A) Weight at 24 h. Weight remained similar between the treatment (271.59 ± 3.58) and nontreatment groups (268.26 ± 3.11; *p* = 0.48), indicating comparable early poststroke status. (B) Weight at 48 h. The treatment group showed significantly higher weight (284.97 ± 3.76) than the nontreatment group (260.72 ± 3.25; *p* = 3.10 × 10^−6^), reflecting preserved physiological integrity. (C) Weight change at 24 h (%). Weight loss was attenuated in the treatment group (92.30% ± 0.82%) compared with the nontreatment group (89.83% ± 0.73%; *p* = 0.03), suggesting an early benefit of treatment on systemic homeostasis. (D) Weight change at 48 h (%). The treatment group maintained markedly higher weight retention (94.85% ± 0.96%) than the nontreatment group (87.29% ± 0.92%; *p* = 9.60 × 10^−8^), demonstrating sustained protection against stroke‐induced physiological decline.

Weight‐related measures displayed mixed but informative results. Body weight at 24 h did not differ between groups (*p* = 0.48), suggesting comparable early postinjury physiological conditions (Figure 8A). In contrast, weight (48 h) was significantly higher in the treatment group (*p* = 3.10 × 10^−6^), and both weight change (%) at 24 and 48 h showed attenuated weight loss (*p* = 0.03; *p* = 9.60 × 10^−8^, respectively).

Importantly, comparison of fold changes revealed that the Comprehensive Score (fold change = 0.762) most closely matched the fold change of the infarct volume (0.765), outperforming all other variables (Table 5). This close alignment further supports the Comprehensive Score as the most faithful surrogate for treatment‐induced structural protection.

**TABLE 5:** Differential analysis results of variables between treatment and nontreatment groups.

Variable	Nontreatment (mean ± SE)	Treatment (mean ± SE)	Fold change	Change direction	*p*
Infarct volume (48 h)	42.24 ± 1.51	32.30 ± 1.16	0.765	Down	2.22 × 10^−9^
Comprehensive Score	2.58 ± 0.11	1.96 ± 0.10	0.762	Down	1.92 × 10^−9^
Infarct Score	2.05 ± 0.10	1.46 ± 0.08	0.712	Down	2.06 × 10^−9^
Longa 5‐point score (0 h)	3.38 ± 0.09	3.69 ± 0.08	1.091	Up	0.02
Belayev 12‐point score (0 h)	8.34 ± 0.18	8.00 ± 0.20	0.959	Down	0.14
Longa 5‐point score (24 h)	2.78 ± 0.16	2.11 ± 0.15	0.760	Down	5.47 × 10^−9^
Belayev 12‐point score (24 h)	7.80 ± 0.20	5.11 ± 0.36	0.655	Down	5.95 × 10^−9^
Longa 5‐point score (48 h)	2.89 ± 0.08	2.49 ± 0.05	0.862	Down	2.13 × 10^−9^
Belayev 12‐point score (48 h)	8.36 ± 0.22	6.54 ± 0.16	0.782	Down	2.42 × 10^−11^
Initial weight	299.03 ± 1.94	300.59 ± 1.72	1.005	Up	0.40
Weight (24 h)	268.26 ± 3.11	271.59 ± 3.58	1.012	Up	0.48
Weight (48 h)	260.72 ± 3.25	284.97 ± 3.76	1.093	Up	3.10 × 10^−6^
Weight change (24 h) %	89.83 ± 0.73	92.30 ± 0.82	1.028	Up	0.03
Weight change (48 h) %	87.29 ± 0.92	94.85 ± 0.96	1.087	Up	9.60 × 10^−8^

*Note:* This table presents mean ± standard error values for each variable in untreated and treated groups, along with fold change and *p*‐values. Infarct volume showed a significant reduction with treatment (fold change = 0.765; *p* = 2.22 × 10^−9^). The Comprehensive Score (fold change = 0.762) and Infarct Score (fold change = 0.712) demonstrated closely aligned reductions, reflecting treatment‐induced structural protection. Neurological deficit scores at 24 and 48 h showed functional improvement, whereas weight‐related measures indicated preservation of systemic condition. The close alignment between infarct volume and Comprehensive Score fold changes supports the Comprehensive Score as the most faithful indicator of treatment‐induced changes in infarct burden.

Together, these results demonstrate that the Comprehensive Score and Infarct Score both sensitively capture treatment effects on infarct severity, with the Comprehensive Score providing the most accurate quantitative reflection of neuroprotection. Neurological scores and weight metrics also reflect treatment benefits, though with greater variability and reduced specificity.

### 3.6 Interrater Reliability of the Infarct Score

Interrater reliability analysis was performed for Infarct Score. The Infarct Score demonstrated excellent agreement among the three independent raters, with an ICC of 0.89 (95% CI, 0.86–0.92; *p* < 0.001). Consistent findings were observed using Kendall's coefficient of concordance (*W* = 0.83, *p* < 0.001), indicating strong concordance among raters (Table 6).

**TABLE 6:** Interrater reliability analysis of the Infarct Score.

Analysis	Coefficient	95% CI	*p*
ICC	0.89	0.86–0.92	6.83 × 10^−105^
Kendall's *W*	0.83	—	1.05 × 10^−19^

*Note:* The table presents the intraclass correlation coefficient (ICC) with its 95% confidence interval and *p*‐value, along with Kendall's coefficient of concordance (*W*) and its *p*‐value. The Infarct Score showed excellent agreement among three independent raters (ICC = 0.89, 95% CI: 0.86–0.92, *p* < 0.001; Kendall's *W* = 0.83, *p* < 0.001).

## 4 Discussion

### 4.1 Summary of Major Findings

This study establishes and validates a rapid, nonstaining visual Infarct Score and a composite Comprehensive Score for estimating infarct volume and stroke severity in the rat MCAO model. The Infarct Score demonstrated the strongest single‐variable correlation with TTC‐measured infarct volume, outperforming neurological deficit scores and systemic indicators. By integrating the Infarct Score with neurological performance at 48 h, the Comprehensive Score further improved predictive accuracy, consistency, and stability across untreated and neuroprotectively treated animals. Importantly, the Comprehensive Score closely mirrored treatment‐induced changes in infarct volume, achieved superior cross‐validation performance, and accurately classified infarct severity with a clearly defined cutoff value.

### 4.2 Methodological Innovation and Reliability

A central methodological contribution of this work is a structurally anchored scoring approach that can be applied rapidly without staining, sectioning, or specialized imaging. This innovation addresses two issues in experimental stroke research, including the following: (1) Functional scores are useful but can be rater‐ and training‐dependent, and (2) so‐called gold standard TTC infarct quantification is widely used but imperfect.

Previous work has shown that neurological scoring systems can achieve reasonable validity and reliability, but reliability improves substantially with structured training, certification, or repeated calibration, highlighting inherent subjectivity and the need for standardization (Taninishi et al. 2016; Bieber et al. 2019; Yu et al. 2019). In this context, the Infarct Score adds value because it is based on high salience macroscopic features such as hemispheric asymmetry or swelling and ischemic pallor, rather than multi‐item behavioral judgments, thereby reducing dependence on nuanced rater interpretation and extensive behavioral testing.

Unlike TTC staining or MRI, the Infarct Score relies on macroscopic dorsal‐brain features, ischemic whitening, and hemispheric swelling that are consistently observable and biologically grounded manifestations of infarct evolution. The strong and reproducible correlation between the Infarct Score and infarct volume across both nontreatment and treatment cohorts supports its reliability as a structural surrogate (Bieber et al. 2019).

Reliability is further strengthened by several aspects of the study design. First, analyses were performed in a large, heterogeneous dataset encompassing multiple ischemic durations and a wide range of neuroprotective interventions, reducing the likelihood that observed associations are model‐ or treatment‐specific. Second, the Infarct Score maintained high predictive value in treated animals, where conventional neurological scores often lose sensitivity to tissue‐level injury. Third, cross‐validation analysis demonstrated not only high predictive accuracy but also low variability, indicating robustness against sampling fluctuation and experimental heterogeneity.

### 4.3 Usefulness Beyond Conventional Behavioral Scores

A key practical strength is that the Infarct Score provides a rapid structural context that neurological scores alone often cannot supply. Previous literature indicates that neurological scoring can be valid and reliable in the acute phase, but its relationship to infarct size is inconsistent and depends on the chosen scale, rater consistency, and experimental conditions (Taninishi et al. 2016; Bieber et al. 2019; Yu et al. 2019; Mi et al. 2024). Our results extend this point by showing that under neuroprotective treatment, neurological metrics may improve while their correlation with infarct volume decreases, making a structural substitute particularly valuable for treatment screening and comparative efficacy studies.

Methodologically, the Comprehensive Score is also highly usable, as it integrates a structural readout with a functional metric at the same time point (48 h), yielding better predictive performance and a closer reflection of treatment‐associated infarct reduction than either domain alone. This aligns with broader preclinical principles that multidomain outcome assessment improves inference and reproducibility, particularly when single readouts are vulnerable to bias or dissociation.

### 4.4 Study Limitation and Considerations

This study was based primarily on the widely used filament MCAO model, with most animals undergoing 2 h of ischemia and all experiments performed under a consistent anesthetic protocol using isoflurane with 70% N_2_O. Although the tMCAO2h/R‐restricted sensitivity analysis supported the robustness of the Infarct Score and Comprehensive Score within a single ischemia‐duration model, the generalizability of the proposed scoring system to other ischemia durations, permanent occlusion models, alternative stroke models, or different anesthetic conditions remains to be established. In addition, all treatment and nontreatment datasets were generated within our research group, and the neuroprotective interventions included in the treatment cohort originated from our laboratory. Although the treatment cohort was incorporated to evaluate score performance under different injury severities and treatment responses, it does not represent independent external validation. Future studies should further validate the scoring system across different ischemia durations, alternative experimental stroke models (Sommer 2017), anesthetic protocols (Mousavi et al. 2022), more interventions for neuroprotection (Zhao et al. 2024), and independent datasets generated by other laboratories. Larger prospective multicenter preclinical studies will be important to further establish the robustness, generalizability, and translational utility of the proposed scoring system.

## 5 Conclusion

In conclusion, this study introduces a reliable, practical, and methodologically innovative approach for infarct assessment in experimental stroke. The Infarct Score provides a rapid and nondestructive structural metric, whereas the Comprehensive Score integrates anatomical and functional information to achieve superior predictive performance and robustness. Together, these tools enhance the accuracy, efficiency, and reproducibility of preclinical stroke evaluation and offer clear methodological and translational value.

## Author Contributions

**Changqing Mu:** visualization, data curation, writing – original draft. **Yuchuan Ding:** writing – review and editing, project administration. **Omar Elmadhoun:** writing – review and editing. **Fengwu Li:** writing – review and editing, project administration. **Xiaokun Geng:** writing – review and editing, project administration, funding acquisition.

## Funding

This work was partially supported by the Key Research Special Project of Beijing Natural Science Foundation (Z240021), the National Natural Science Foundation of China (no. 82271332), the Science and Technology Plan of Beijing Tongzhou District (KJ2023CX022), the Beijing Tong Zhou District Financial Fund (2025), and the Yunhe Talent Program of Beijing Tongzhou District (2025).

## Conflicts of Interest

The authors declare no conflicts of interest.

## Supporting information


**Table S1:** Sensitivity analysis of Comprehensive Score and clinical variables with TTC‐derived infarct volume in the tMCAO2h/R subgroup.

## Data Availability

Data will be made available on request.
