# Supplementary material for: Rapid Assessment of Stroke Severity: Development of a Visual Infarct and Comprehensive Scoring System in Ischemic Rats With Middle Cerebral Artery Occlusion
Source: Eur J Neurosci. 2026 Jul 22;64(2):e70611. doi: 10.1111/ejn.70611 (PMC13391210; doi:10.1111/ejn.70611)
Supplement: Supplementary file 1 — Table S1: Sensitivity analysis of Comprehensive Score and clinical variables with TTC‐derived infarct volume in the tMCAO2h/R subgroup. [file EJN-64-0-s001.docx]

**Table S1. Sensitivity Analysis of Comprehensive Score and Clinical Variables with TTC-Derived Infarct Volume in the tMCAO2h/R Subgroup.**

| **Variable** | **Rho** | **p Value** | **Adjusted p Value** |
| --- | --- | --- | --- |
| Comprehensive Score | 0.75 | 1.84 × 10⁻¹⁸ | 2.39 × 10⁻¹⁷ |
| Infarct Score | 0.71 | 1.22 × 10⁻¹⁷ | 1.58 × 10⁻¹⁶ |
| Belayev 12-Point Score (24 h) | 0.59 | 3.07 × 10⁻⁶ | 3.99 × 10⁻⁵ |
| Longa 5-Point Score (48 h) | 0.51 | 5.53 × 10⁻¹⁸ | 7.19 × 10⁻¹⁷ |
| Longa 5-Point Score (24 h) | 0.47 | 6.15 × 10⁻⁵ | 8.00 × 10⁻⁴ |
| Belayev 12-Point Score (0 h) | 0.25 | 0.04 | 0.56 |
| Belayev 12-Point Score (48 h) | 0.22 | 2.01 × 10⁻^3^ | 0.03 |
| Longa 5-Point Score (0 h) | 0.01 | 0.93 | 1.00 |
| Initial Weight | -0.13 | 0.12 | 1.00 |
| Weight (24 h) | -0.20 | 0.05 | 0.70 |
| Weight Change (24 h) (%) | -0.34 | 1.12 × 10⁻^3^ | 0.02 |
| Weight (48 h) | -0.37 | 7.56 × 10⁻⁶ | 9.82 × 10⁻⁵ |
| Weight Change at (48 h) (%) | -0.41 | 6.55 × 10⁻⁷ | 8.51 × 10⁻⁶ |

This table presents Spearman correlation coefficients (Rho), p values, and adjusted p values for the Comprehensive Score and 12 variables in a sensitivity analysis restricted to the tMCAO2h/R subgroup. The Comprehensive Score demonstrated the strongest association with infarct volume (Rho = 0.75, adjusted p = 2.39 × 10⁻¹⁷), followed by the Infarct Score (Rho = 0.71, adjusted p = 1.58 × 10⁻¹⁶). Overall, the correlation patterns remained consistent with the primary analysis, indicating that the associations between the proposed scoring system and TTC-derived infarct volume were preserved after restricting the analysis to a single ischemia-duration model.
